# Facial impression of trustworthiness biases statement credibility unless suppressed by facemask

**DOI:** 10.1007/s12144-022-03277-7

**Published:** 2022-06-09

**Authors:** Marco Marini, Fabio Paglieri, Alessandro Ansani, Fausto Caruana, Marco Viola

**Affiliations:** 1 Institute of Cognitive Sciences and Technologies (ISTC), Italian National Research Council (CNR), Rome, Italy; 2 Department of Psychology, Sapienza University of Rome, Rome, Italy; 3 Cosmic Lab, Department of Philosophy, Communication, and Performing Arts, Roma Tre University, Rome, Italy; 4 Institute of Neuroscience, Italian National Research Council (CNR), Parma, Italy; 5 Department of Philosophy and Education, University of Turin, Turin, Italy

**Keywords:** Face perception, First impressions, Implicit bias

## Abstract

The impression of trustworthiness based on someone’s facial appearance biases our subsequent behavior toward that subject in a variety of contexts. In this study, we investigated whether facial trustworthiness also biases the credibility of utterances associated with that face (H1). We explored whether this bias is mitigated by utterances eliciting reasoning, i.e. explanations (as opposed to factual statements; H2). Moreover, we hypothesized that overimposing facemasks on those faces could enhance/reduce utterance credibility due to social value of mask-wearing (H3), and that facemasks could counter the putative credibility bias introduced by facial trustworthiness (H4). If so, this may be either because facemasks remove the visual information necessary for trustworthiness impression (H4a), or because information is less salient, although it can be retrieved under different circumstances (H4b). An online study (N = 159) was conducted to test these hypotheses. In the first task, subjects saw 48 facial pictures coupled with one utterance and judged the truthfulness/falsity of this utterance. In the second task, they saw again 16 of the faces from the previous tasks and were asked to recall whether the associated utterance was true or false. Findings from the first task support H1 and H4, but not H2 and H3. However, in the second task, where the face is the only available cue, the credibility-mitigation bias exerted by facemask disappears, supporting H4b over H4a. Our results confirm the pervasivity of facial trustworthiness impressions in social cognition, and suggest that facemask can mitigate them, or at least their salience.

**Supplementary information:**

The online version contains supplementary material available at 10.1007/s12144-022-03277-7.

## Introduction

How do we judge the reliability of someone’s statement? When we have enough time, we can verify it thoroughly by comparing multiple sources. However, this time-consuming process can rarely be implemented in real-life situations. Most times, we cannot but settle for estimating the credibility of the utterer. When we meet someone that we already know, our previous biographical knowledge provides us with an answer to the question “shall I trust her?”. However, real-life is replete with situations where we must judge the validity of statements uttered by a stranger. In such cases, one may argue that our choices can be driven by first impressions, especially those based on facial appearance.

A vast literature shows that first impressions from faces are surprisingly consistent across subjects (Todorov et al., 2015; but see Hehman et al., 2019). The process of impression formation from faces is automatically triggered by face-looking stimuli (i.e., objects resembling an upside-down triangle gestalt, “∵”), and requires very little time – often less than 100 ms (Willis & Todorov, 2006; Todorov et al., 2009). Impression formation is often implicit, i.e. it can work despite, and even against, our conscious beliefs. Thus, some scholars (e.g. Todorov et al., 2015; Bonnefon et al., 2013) have compared the mechanisms for impression formation with mental modules (Fodor, 1983), domain-specific mechanisms that quickly and automatically perform some cognitive operation independently from conscious cognition, whereas Todorov (2017: 57) presents impression formation in terms of Kahneman’s “System 1”. In Kahneman’s interpretation of the dual-process framework, by default most cognitive processes are carried out by System 1 (or fast thinking), which refers to cognitive processes that (similarly to modules) yield fast and frugal heuristic solutions, often without conscious access nor control. On the contrary, System 2 (or slow thinking), involving conscious thinking, is slower and more accurate, allowing for less stereotyped processes, but is also “lazy”, i.e. it becomes active only when a specific task demands it.

Most of these judgments have been shown to load onto common underlying dimensions, namely *trustworthiness* and *dominance* (Oosterhof & Todorov, 2008). Indeed, even though several authors stress their misleading nature (Porter et al., 2008; Olivola & Todorov, 2010; Bonnefon et al., 2013), facial trustworthiness judgments have been found to reliably predict trustors’ behavior in a hoard of different contexts, such as trial outcomes (Porter et al., 2010; Wilson & Rule, 2015), medical triage (Bagnis et al., 2020), and disposition to act cooperatively in the face of a natural hazard (Felletti & Paglieri, 2019).

While a significant amount of evidence has been gathered documenting how facial trustworthiness impression can bias behavioral choices, to what extent this feature affects the reliability of someone’s statement is surprisingly understudied. Thus, when someone does not *know* whether some statement is true or whether some explanations are valid, we hypothesize that (H1) impressions of utterer’s facial trustworthiness affect the credibility of the utterances.

However, if such a bias obtains, we also speculate that (H2) it might affect factual statements (henceforth simply ‘facts’; e.g., “Tokyo is the most populous city in the world”) more severely than explanations (e.g., “The oceans release large amounts of oxygen into the atmosphere due to the movement of sea currents”). If the influence on credibility of impression of trustworthiness based on facial appearances is indeed a manifestation of “fast thinking” strategies to evaluate sentences, explanations, by their own nature, might prompt instead engagement of System 2 mechanisms, thereby triggering “slow thinking” strategies to assess the utterance and attenuating or canceling the biasing effect of faces.

Another factor possibly mitigating the effect of trustworthiness bias is the partial facial occlusion. Indeed, capitalizing on the introduction of norms for mask-wearing in response to the Covid pandemic, several studies have recently investigated how facemasks influence the formation of the impression of trustworthiness (Cartaud et al., 2020; Olivera-La Rosa et al., 2020; Biermann et al., 2021; Grundmann et al., 2021; Malik et al., 2021; Marini et al., 2021; Oldmeadow & Koch, 2021). The results of these studies suggest that masks can affect trustworthiness in two ways that are not mutually exclusive. First, by preventing visual access to the lower part of the face, masks can filter out some visual cues of trustworthiness. However, facemasks are not like any other face coverings (Calbi et al., 2021): as they have been imbued with specific and contextual social meanings, mask-wearing can also be interpreted as a signal that enhances (or lowers) the reliability of those who don it.

If this social interpretation occurs, (H3) an increase (or decrease) of utterances credibility should be seen as a result of mask-wearing itself, independently of face trustworthiness. Whereas if mask only interacts with credibility by filtering out facial details for trustworthiness estimation, we should expect that (H4) masks affect statements credibility by mitigating the facial trustworthiness bias.

If this filtering effect obtains, this can be due to two slightly different mechanisms. On a simple view, (H4a) facial cues of trustworthiness simply become unavailable. But another explanation is that, by obstructing the lower part of the facial gestalt (∵) that triggers the facial impression formation module, facemasks may *suppress* the automaticity of its activation. If this obtains, we should expect that (H4b) facial trustworthiness does not override other sources of information if they are available but can still be accessed when they are not.

To investigate the hypotheses listed above, we ran an online behavioral study on a sample of Italian subjects. The experiment was composed by two tasks. In order to prevent the participants from looking for the answers, both tasks were run under time constraints. In the first task, subjects have to assess the truth- or false-hood of some facts or explanations, presented together with a face picture. Subjects were split in two groups, one seeing only unmasked face pictures, the other seeing the very same pictures with mask (Fig. 2).

If trustworthiness does in fact bias the credibility of utterances, high and low trustworthy-looking faces should respectively raise or decrease the rate of “true” responses associated with them (H1). Explanations may be less affected or unaffected by this bias than facts, because they may foster some “slow thinking” strategy that mitigates it (H2).

**Fig. 1:**
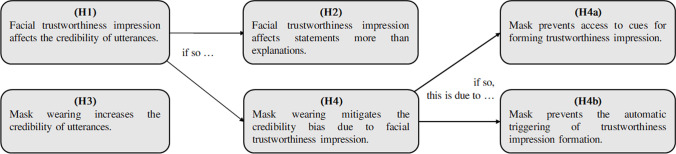
A visual summary of the six hypotheses of this study

Assuming that (H1) is correct, then if masks are interpreted as a positive (or negative) social signal of (un)reliability (H3), subjects who see masked faces should respond “true” (or “false”) more often than those who see unmasked faces, independently of the stimuli normative trustworthiness. If facemasks dampen the credibility-enhancing bias of trustworthy faces (H4), or the credibility-decreasing bias of untrustworthy ones, this can be either due to the unavailability of cues for forming the trustworthiness impression (H4a), or to a suppression of the mechanism that automatically promotes the use of this impression (H4b) (See Fig. 1 for a visual summary of the six hypotheses).

To disambiguate between these two mechanisms, in the second task, subjects were shown a subset of the face pictures they saw in the first part and were asked to recall whether the statement uttered by each face was correct or incorrect. In fact, while in the first task subjects could try to guess the plausibility based on verbal information, the second task worked as a control condition in which facial impression was the only visual information available.

## Methods

### Participants

The experiment was an online test carried out on 180 Italian native speakers, recruited by means of different social media platforms (i.e., snowball procedure). The participation was on a voluntary basis, and no payment or reward was provided to the participants. Data collection was made in May 2021. Before the experiment, participants read the main instructions and provided informed consent, while, after the experiment, they provided some basic demographic information. By accessing a single un-reusable link, each participant could run the experiment directly from home on their laptops, smartphones, or tablets. An anti-ballot box stuffing was employed in order to avoid multiple participations from the same device. The study was conducted in accordance with the ethical standards laid down in the 1964 Declaration of Helsinki and was granted ethical approval by the Institutional Review Board (IRB) of Sapienza University Rome (ID 0001448 - 5.7.2021 [UOR: SI000030-Classif. II/23).

After data collection, 21 participants were excluded according to the following pre-established, exclusion criteria: (a) participants who did not finish the test (n = 13); (b) participants who completed the task in more than 3SD over the average duration (n = 5); (c) participants who selected the same option for > 90% of the times (n = 3). The remaining 159 participants (95 females; age = 31.07 ± 8.34) successfully completed the task and were included in all the analyses. This number was deemed sufficient according to an a-priori power analysis with G*Power (version 3.1.9.7; (Faul et al., 2007)), who suggested that a sample size of n = 124 was required to identify a difference in credibility scores between masked and unmasked faces and trustworthy and untrustworthy ones. We run the a-priori power analysis assuming a repeated-measures ANOVA, within-between interaction (trustworthiness*credibility), power = 0.95, α error probability = 0.05. As for the effect size, we hypothesized one similar to that of a previous study (Marini et al. 2021) using a similar task (partial η^2^ = 0.026).

### Stimuli

#### Faces

**Fig. 2:**
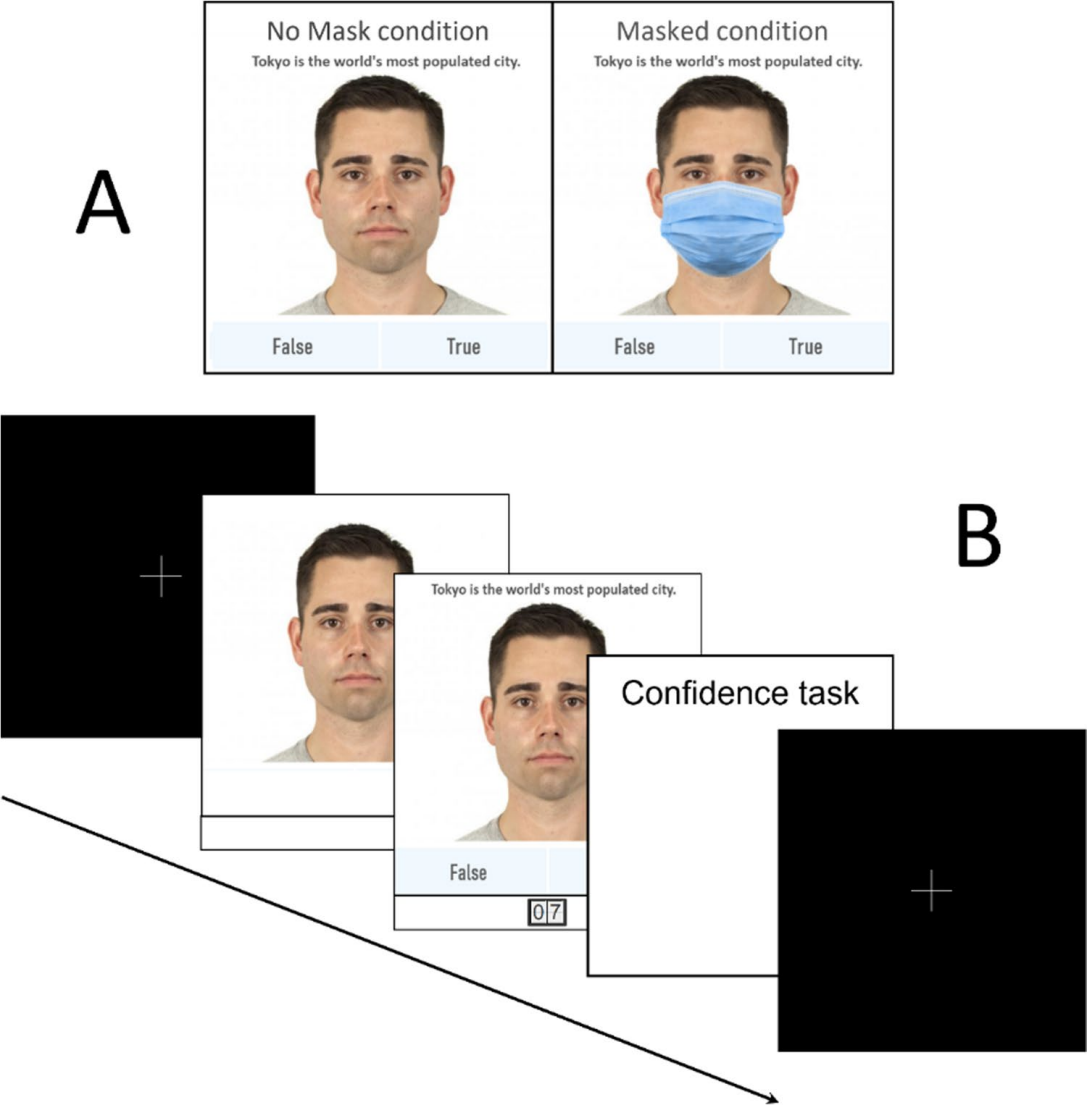
Experimental procedure. **A **stimuli consisted of faces from Chicago Face Database (CFD) presented in a masked or unmasked fashion. **B **In the first session, participants were presented with CFD stimuli; after a 3-second exposure, a statement and a countdown appeared. Participants were required to judge the veracity of the statement. Subsequently, the confidence rating was submitted via a 4-point Likert scale before a new fixation cross introduced a new stimulus

Face stimuli (n = 48) were selected from the Chicago Face Database (CFD; Ma et al., 2015), considering 12 high-trustworthy and 12 low-trustworthy faces for each gender, on the basis of trustworthiness scores. The selection was limited to white faces, reflecting the most represented ethnicity of the participants’ nationality. Each face displayed a neutral expression. Stimuli were edited by a professional graphic designer by superimposing a standard surgical mask (see Fig. 2), to finally obtain the same set of stimuli in both masked and unmasked versions. The original pictures used in this study are publicly available. For the complete list of the stimuli, please see the Supplementary Materials.

#### Statements

A second set of stimuli consisted of 48 statements (in Italian), reporting general facts (n = 24) and explanations (n ​​= 24). For each category of statements, information was either true or false in equal measure. To identify statements whose truthfulness was guessed at the chance level, statements were selected based on a preliminary pilot (N = 24) conducted on 80 original statements. The average percentage of “true” responses of the 48 chosen stimuli was 50% (SD = 18.4%). None of the employed statements reported an accuracy above 70% (stimuli had to be ambiguous, neither clearly true nor evidently false).

Statements reporting general facts communicated factual information (either true or false), whereas statements reporting explanations were always constituted by true information, accompanied by either a true or a false explication. Examples of facts and explanations are the following: “*The left lung is on average greater than the right by 10%*” (true fact); “*During the summer the weather is warmer because the Earth is closer to the Sun*” (false explanation; for the complete list of the statements, please see the Supplementary Materials). All statements were comparable in length (mean length: 113 characters; SD = 17.36) and related to different topics from both humanities (history, geography, and arts) and scientific (natural science and biomedicine) domains in a balanced fashion.

During the experiment, each statement was uniquely associated with a face (see below). Information type (facts and explanations), veracity (true or false), and domains were balanced for gender and trustworthiness of the face associated with each statement.

### Experimental Procedure

The study was an online test carried out on Qualtrics.com®. Upon acceptance of the informed consent, each participant was randomly assigned to one of the two groups (i.e., masked, N = 79; or unmasked conditions, N = 80). In both conditions, the experiment was divided into two consecutive blocks:

1. Block 1: Credibility and confidence task. Each trial started with a fixation cross, lasting 1000ms and then followed by the presentation of a face. After 3000ms, the presented face was flanked by (a) a written statement, appearing in the upper part of the screen, above the face, and (b) a 10-second countdown, visible in the lower part of the screen, below the face. During the 10-second countdown, participants were requested to judge whether the statement was reporting true or false information. Once the answer was given, a new screen displaying the same face inquired the participants’ confidence in their previous answer on a 4-point Likert scale (see Fig. 2).
2. Block 2: Recall task**.** Sixteen random faces from the previously presented set (counterbalanced for face trustworthiness and statements veracity) were shown to the participants, one at a time and in random order. The task was to judge whether the presented face had previously communicated true or false information. The response was given via a 4-point Likert scale, ranging from definitely false to definitely true, presented below the presented face. Unlike Block 1, there were no time constraints or any other information. A new trial began after the answer was given.

### Statistical Analysis

Analyses were conducted by means of IBM SPSS 26.0. The power of the test was set at 90% and the significance level was set to α = 0.05. All variables were checked for normality by the Shapiro-Wilk test and for homoscedasticity by the Levene test. When violating the above assumptions, data normality was assessed via skewness and kurtosis for medium-sized samples (Kim, 2013; George & Mallery, 2010). When the distribution of the sample was non-normal, non-parametric tests were applied. Post-hoc pairwise comparisons were adjusted by means of Bonferroni correction and were investigated only upon a previous significant main effect. In the graphs of this article, error bars represent 95% confidence intervals. Anonymized data are publicly available at: https://osf.io/9jmqg/?view_only=303d8fa0247340909ec165a6d0bd8c74.

#### Block 1. Credibility and Confidence

##### Credibility

The first analysis was aimed at assessing the number of times a participant considered a statement to be true, regardless of its actual correctness. This issue was investigated by a four-way mixed ANOVA considering Maskedness (*masked/unmasked* as a between-subjects factor), Face Trustworthiness (*trustworthy/untrustworthy*), Information Type (*facts/explanations*), and Veracity (*true/false*) as main factors (all within-subject factors), applied to the credibility scores.

##### Response Times (RTs)

The analysis was performed on all RTs satisfying the following criteria: (a) RT above a cut-off threshold of 3 s for each choice and (b) RT within 3 SDs from the trial per subject mean. We parameterized the RTs by subtracting to each of them the individual mean RT. This procedure allowed us to have positive values when the RT of a given category of stimuli was above the average RT of the subject for the whole task, and negative values when RTs were below the average RT. As all factors have two levels, the scores of the parameterized RTs for each level of the variable are the same (indeed, their mean is 0), only changing in their sign. For this reason, in this analysis, we merely reported the mean difference (MD) and the relative effect size. Finally, we run a four-way mixed ANOVA considering Maskedness (*masked/unmasked* as a between-subjects factor), Face Trustworthiness (*trustworthy/untrustworthy*), Information Type (*facts/explanations*), and Veracity (*true/false*) as main factors.

##### Confidence

A third analysis was aimed at assessing the participants’ confidence in the given answer, as measured by a 4-point Likert scale. Similarly to the previous analysis, we run a four-way mixed ANOVA considering Maskedness (*masked/unmasked* as a between-subjects factor), Face Trustworthiness (*trustworthy/untrustworthy*), Information Type (*facts/explanations*), and Veracity (*true/false*) as main factors (all within-subject factors), applied to the confidence score.

#### Block 2. Recall Task

To investigate the extent to which the participant believed each face to have previously provided true information, and to finally evaluate the impact of face trustworthiness and maskedness on recall scores, we ran a two-way mixed ANOVA considering Maskedness (*masked/unmasked*) and Face Trustworthiness (*trustworthy/untrustworthy*) as main factors, applied to the score obtained in the recall task by the 4-point Likert scale.

#### Preliminary Sample Analysis

Before proceeding with the main analyses, we ensured that our experimental groups were balanced in terms of possible socio-demographic intervening factors (i.e., professional, or educational biases). A preliminary ANOVA reported no difference in mean age, educational level, and type of employment between the masked and unmasked groups (all ps > 0.34). Secondly, to avoid an unbalance in the groups’ expertise, the general performance of our participants was controlled. More specifically, we did not find any significant difference in the accuracy (i.e., the ability to recognize the veracity of the employed utterances) between the two experimental groups (all the utterances’ domains reported p > .09).

## Results

### Statement Credibility

**Fig. 3:**
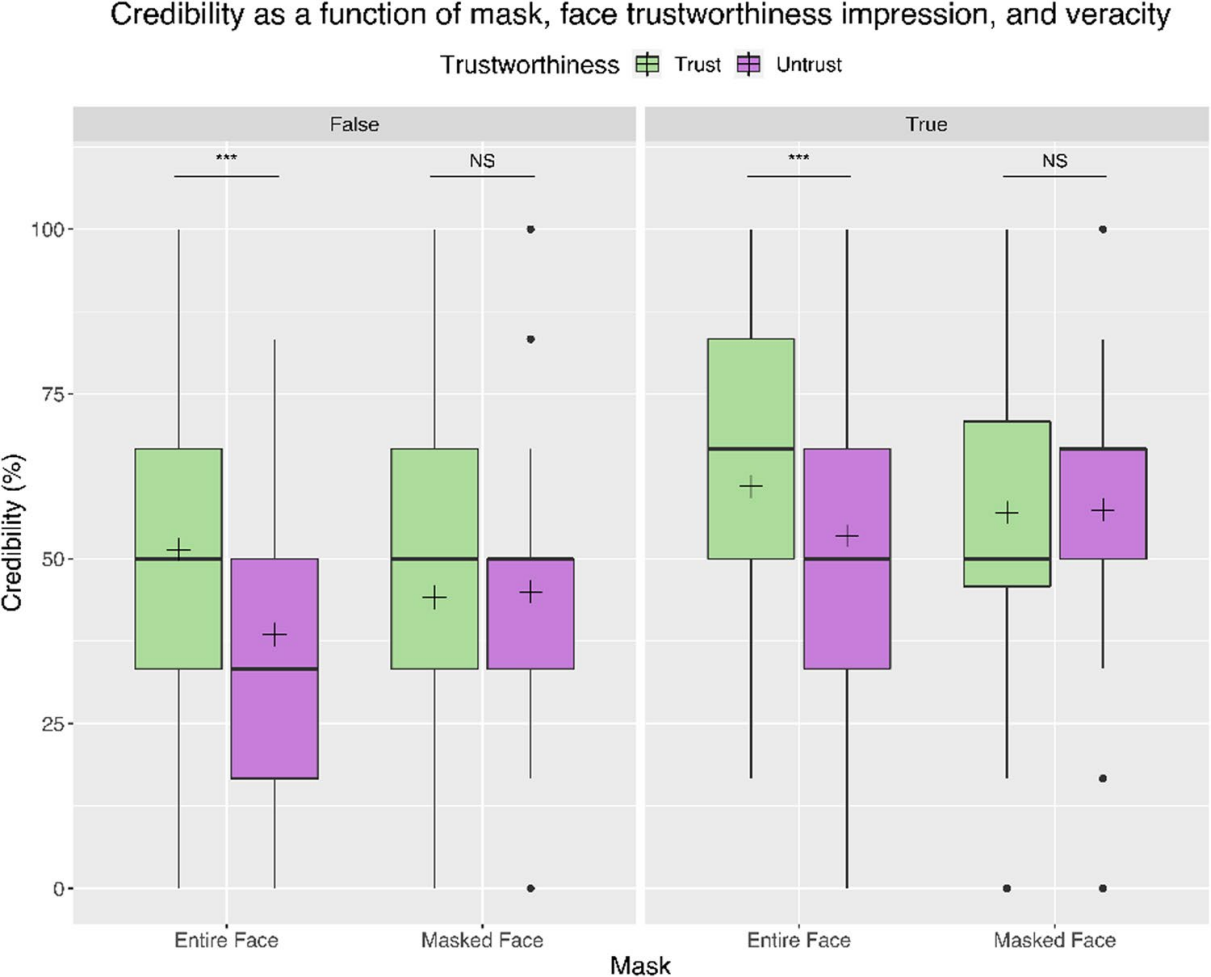
Credibility as a function of mask, face trustworthiness impression, and veracity. The boxplots represent interquartile ranges (IQRs). Black horizontal lines within the boxplots indicate median values; black crosses represent mean values. Horizontal bars and asterisks indicate significant results (***p < .001). Black points mark outliers. The graph shows the significant interaction between maskedness and face trustworthiness, that is, the credibility scores vary in dependence of the trustworthiness impression only in the unmasked condition

The first analysis showed a significant main effect of the Face Trustworthiness (F(1,157) = 20.75, p < .001, η^2^p = 0.12), with trustworthy faces inducing more credibility in statements than untrustworthy faces do (M = 53.37% SE = 1.13% and M = 48.59% SE = 0.99%, respectively). We also found a main effect of Veracity (F(1,157) = 94.88, p < .001, η^2^p = 0.38), while both Maskedness (p = .92) and Information Type (p = .72) failed to show significant results (Fig. 3).

While Maskedness failed to show a significant main effect per se, we found a significant interaction between Maskedness and Trustworthiness (F(1,157) = 26.53, p < .001, η^2^p = 0.15). More specifically, when the statement was associated to an unmasked face, face trustworthiness increased credibility judgments (trustworthy M = 56.17% SE = 1.60%; untrustworthy M = 46.00% SE = 1.40%; p < .001). However, when the face was covered by a facemask (p = .67; Fig. 3), face trustworthiness failed to affect credibility judgments, in line with the hypothesis that trustworthiness drove the participants’ answers only when the face is fully visible (H1), while information on face trustworthiness was mitigated by facemasks (Fig. 3).

In addition, we found a significant interaction between Veracity and Information Type (F(1,157) = 146.46, p < .001, η^2^p = 0.48), showing that true explanations are considered more credible than false ones, whereas there is no difference in credibility between true and false facts; this indicates that subjects were more accurate in answering to explanations than to facts.

### Response Times (RTs)

**Fig. 4:**
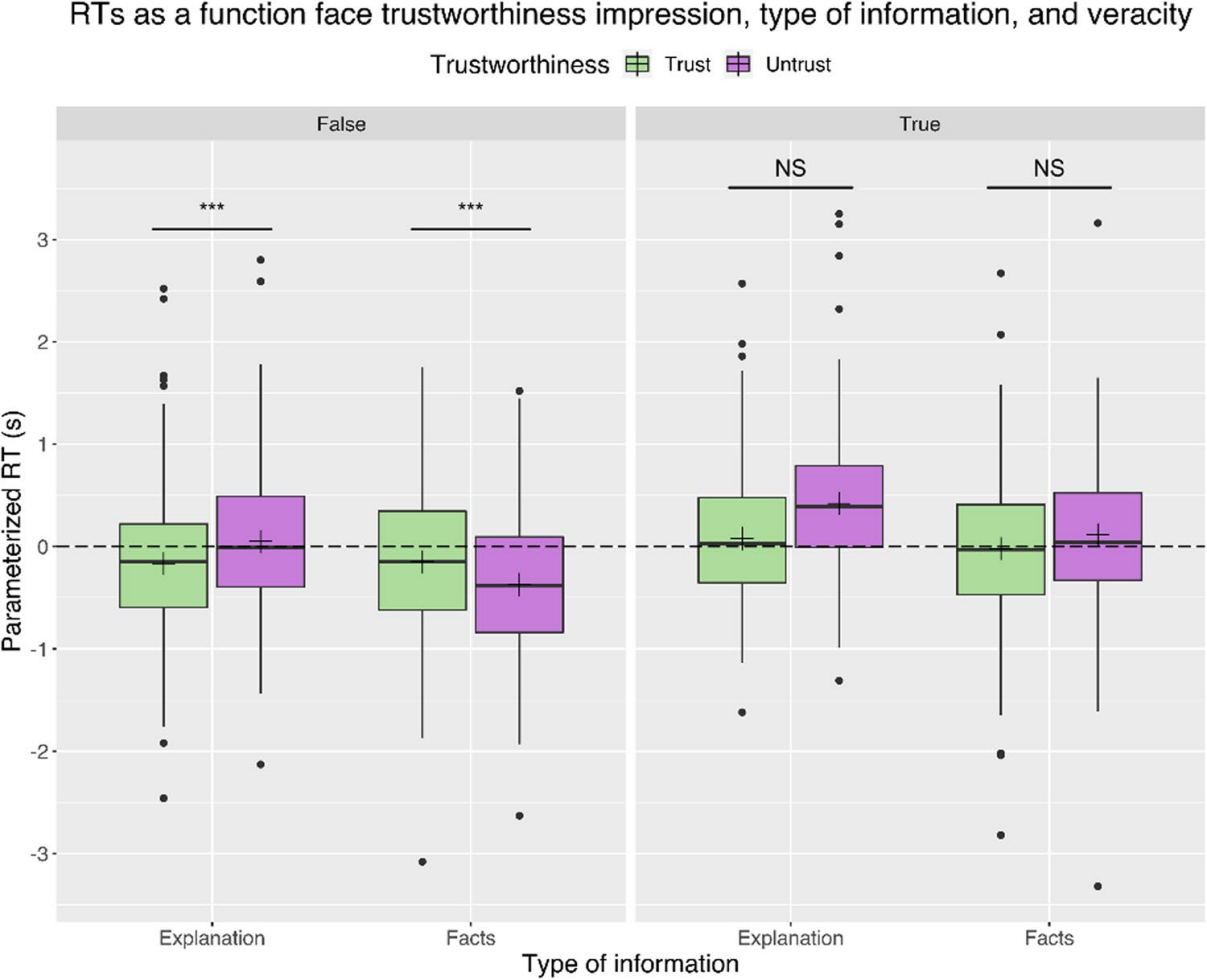
Parameterized RTs as a function of face trustworthiness impression, type of information, and veracity. The boxplots represent interquartile ranges (IQRs). Black horizontal lines within the boxplots indicate median values; black crosses represent mean values. Black points mark outliers. Horizontal bars and asterisks indicate significant results (***p < .001). The horizontal dashed line represents the average RT: values above zero indicate RTs longer than the subjects’ mean. Participants took longer to respond to explanations, true statements, and faces perceived as untrustworthy

The analysis of RTs relative to the credibility task showed a significant main effect of Face Trustworthiness (F(1,157) = 9.81, p = .002, η^2^p = 0.06), showing longer RTs for untrustworthy as opposed to trustworthy (MD = 0.13 s SE = 0.04) faces (Fig. 4). In addition, we found a main effect of Information Type (F(1,157) = 22.69, p < .001, η^2^p = 0.13), with longer RTs for explanations (MD = 0.22 s SE = 0.04), and a main effect of Veracity (F(1,157) = 40.21, p < .001, η^2^p = 0.20), with longer RTs for true statements (MD = 0.30 s SE = 0.05) (Fig. 4). Maskedness failed to show significant results (p = .55).

### Confidence

The study of the confidence reported by participants in giving their judgment showed a main effect of Face Trustworthiness (F(1,157) = 6.23, p = .014, η^2^p = 0.04) with trustworthy faces inducing more confidence in statements than untrustworthy faces. More specifically, trustworthy faces scored higher in confidence (M = 2.61 SE = 0.03) compared to untrustworthy ones (M = 2.56 SE = 0.03) (see Fig. A in the Supplementary Materials).

In addition, we also found a significant effect of Information Type (explanations: M = 2.47 SE = 0.03; facts: M = 2.70 SE = 0.03) F(1,157) = 135.03, p < .001, η^2^p = 0.46), and Veracity (false M = 2.52 SE = 0.03;truth: M = 2.65 SE = 0.03) F(1,157) = 35.49, p < .001, η^2^p = 0.18) (see Fig. A in the Supplementary Materials). Similar to the case of credibility, the main effect of Maskedness was not significant (p = .22).

We also found a significant interaction between Face Trustworthiness and Veracity (F(1,157) = 17.01, p < .001, η^2^p = 0.10), revealing that, when associated to true statements, trustworthy faces induced more confidence than untrustworthy ones (M = 2.60 SE = 0.03 vs. M = 2.44 SE = 0.03, p < .001) and, symmetrically, when associated to false statements, untrustworthy faces induced more confidence than trustworthy ones (M = 2.68 SE = 0.03 vs. M = 2.60 SE = 0.03; p = .001). Moreover, as in the case of credibility, we found a significant interaction between Information Type and Veracity (F(1,157) = 17.01, p < .001, η^2^p = 0.10), showing that confidence is higher for explanations than for facts (consistently with the greater accuracy exhibited with the former type of statements), although this effect is more marked for true statements than for false ones (see Fig. A in the Supplementary Materials).

### Recall Task

**Fig. 5:**
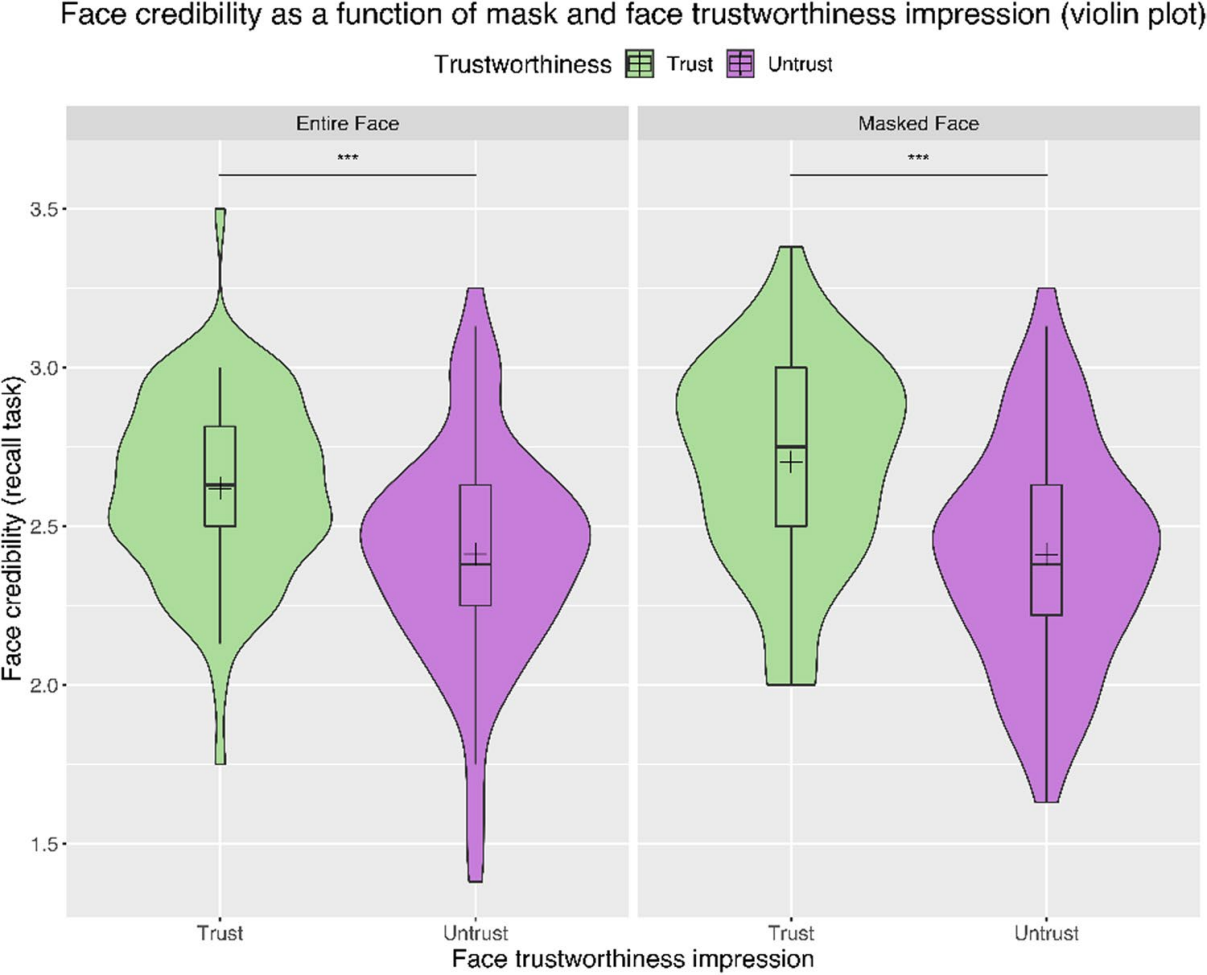
Face credibility as a function of mask and face trustworthiness impression during the recall task (violin plot). The form of the violin indicates the distribution curve. The boxplots within each violin represent interquartile ranges (IQRs). Black horizontal lines within the boxplots indicate median values; black crosses represent mean values. Horizontal bars and asterisks indicate significant results (***p < .001). Regardless of the presence of the mask, face credibility scores were coherently associated with face trustworthiness impression

The study of the recall task showed a main effect of Face Trustworthiness (F(1,157) = 40.89, p < .001, η^2^p = 0.21), with trustworthy faces more associated with true statements than untrustworthy ones (M = 2.66 SE = 0.04 vs. M = 2.41 SE = 0.03) (Fig. 5). We did not find a significant effect of Maskedness (p = .23), nor a significant interaction (p = .29), indicating that the same pattern was stable in both conditions and that participants were able to visually discriminate between trustworthy and untrustworthy faces also in the masked condition (for a summary of all results, see Table 1).

**Table 1:** Main effects and interactions results of the three experimental tasks

		Credibility		Confidence		Recall	
		p-value	direction	main effect	direction	p-value	direction
Main effects	Maskedness	> 0.05	-	> 0.05	-	> 0.05	-
	Trustworthiness	< 0.001	Trust > Untrust	< 0.001	Trust < Untrust	< 0.05	Trust > Untrust
	Information type	> 0.05	-	< 0.001	Expl.>Facts	< 0.001	Expl.>Facts
	Veracity	< 0.001	True > False	< 0.001	True > False	< 0.001	False > True
Interactions	Maskedness * Trustworthiness	< 0.001		> 0.05		> 0.05	
	Maskedness * Information type	> 0.05		> 0.05			
	Maskedness * Veracity	> 0.05		> 0.05			
	Trustworthiness * Information type	> 0.05		> 0.05			
	Trustworthiness * Veracity	> 0.05		< 0.001			
	Information type * Veracity	< 0.001		< 0.001			

## Discussion

We conducted a study showing that facial trustworthiness impressions can bias the credibility of statements associated with them (H1), regardless of the specific type of statement (explanations or facts; H2). We also investigated whether a sentence uttered by a person wearing a facemask can be perceived as more (or less) reliable because of the signal value of the mask, finding that, while not exerting a direct effect on statements credibility (H3), facemasks indirectly affect statements credibility by mitigating the facial trustworthiness bias (H4). Finally, we disambiguated between two putative mechanisms, finding that masks did not prevent the access to visual information for trustworthiness estimation (H4a), but prevented the automatic triggering of facial trustworthiness bias (H4b). These findings will be discussed in turn below.

Let us begin with (H1). In the credibility and confidence task, we found that (unmasked) faces with above-average trustworthiness scores consistently inflate the credibility of statements paired with them, whereas faces with below-average scores decrease them. Subjects’ responses were also faster and associated with higher confidence ratings in the presence of trustworthy faces. Moreover, in our second task, when asked to recall whether a given face was associated with a true or a false statement, subjects consistently ascribed more true statements to trustworthy than to untrustworthy faces. Both results concur in supporting H1.

Other than with intuition, our study is in line with the vast literature attesting that facial trustworthiness impression occurs quickly (Todorov et al., 2009; De Neys et al., 2017; Jessen & Grossmann, 2019) and affects the trustor’s behavior toward the trustee in several contexts, spanning from trials outcome (e.g., Wilson & Rule 2015) to medical triage (Bagnis et al., 2020. For a review, Todorov et al., 2015). However, while several studies have documented the effect of trustworthiness impression on decisions, to the best of our knowledge, this is the first evidence that trustworthiness impression also affects the credibility of statements.

Contrary to our prediction, both facts and explanations are biased by facial trustworthiness (H2). The message features that we manipulated had only limited effects on participants’ performance during the sentence judgment task. We speculated that judgments concerning explanations might be less affected than those on facts by facial impression biases because their logical structure could mobilize some “slow thinking” strategy that overrides the facial trustworthiness heuristic, a notable “fast thinking” process (Todorov, 2017: 57). Interestingly, the evidence we found suggests that explanations manage to elicit slow(er) thinking, as reflected by slower reaction times and higher confidence in judgments of explanations than in those of facts, notwithstanding the comparable length of either type of sentence. However, as slower responses were not associated with a reduction of bias, we found no support for H2, at least under the time constraints of the present study. Together with other evidence of the stubbornness of facial trustworthiness biases coming from a recent study showing its recalcitrance to go away despite specific training (Jaeger et al., 2020), the difficulty of slow thinking to override the impression cast doubts over the appropriateness of conceiving facial impressions in terms of dual theories à la Kahneman (2011).

What modulates statements’ credibility instead are facemasks. Extant studies focus on either explicit judgments or implicit measures of trustworthiness impressions from masked faces (Cartaud et al., 2020; Olivera-La Rosa et al., 2020; Biermann et al., 2021; Grundmann et al., 2021; Malik et al., 2021; Marini et al., 2021; Oldmeadow & Koch, 2021) have highlighted two ways in which masks can act upon the trustworthiness impression of a face who don it. First, the very presence of a facemask can enhance trustworthiness impression, arguably due to signaling care for others (Cartaud et al., 2020; Marini et al., 2021; Olivera-La Rosa et al., 2020). In some cases, facemasks have also been reported to decrease trustworthiness impression, likely due to their association with the harm brought about by the Covid-19 pandemic (Biermann et al., 2021; Malik et al., 2021). In both cases, however, the effect is due to the meaning assigned to the mask itself rather than on its effect on the underlying face. Hence, it should show up regardless of the masked face’s normative trustworthiness, as predicted by our hypothesis H3. Our results do not support this hypothesis, as we did not observe any main effect of the mask on the results of credibility of statements during the second task nor of the responses in the recall task.

Another possible interaction between facemask and trustworthiness may obtain, i.e., that facemasks can mitigate positive and negative biases due to a face’s perceived trustworthiness – as per H4. If so, the average credibility of statements should be uncorrelated with masked faces’ trustworthiness, unlike that of statements correlated with unmasked faces. Our data provide support for this hypothesis, thus suggesting that facemasks mitigate facial trustworthiness bias.

This finding seems challenged by a recent study (Twele et al., 2022, study 1a). In their study, no significant difference in explicit trustworthiness ratings of faces was found between the unmasked and masked conditions. The discrepancy between the results of our credibility assessment task and Twele and colleagues’ explicit judgment task can be due to some difference in task demands.

Two mechanisms can account for why facemasks mitigate the facial trustworthiness bias. The simpler explanation is that a facemask prevents visual access to facial cues that are crucial for forming a trustworthiness impression (H4a). However, this hypothesis seems at odds with the results of the recall task, in which subjects reported that un/trustworthy faces were less/more likely to have uttered a true statement during the first task, irrespectively of the presence of a mask.

An alternative account maintains that the visual information underlying trustworthiness impression is still available even in masked faces. On this account, facemasks suppress the automatic triggering of impression formation, but this impression can be restored if the face is attended to deliberately (H4b). Accordingly, in our first task, facemasks block the facial trustworthiness bias because visual attention is diverted by the statement, whereas in our recall task, facial trustworthiness bias is restored because, lacking other visual cues, the faces are attended to. And the same goes, we surmise, for Twele and colleagues’ task, where subjects were explicitly required to attend to faces.

This suppression effect is inspired by, and consistent with, well-established findings regarding the automaticity of face detection in neurotypical humans. We know that face-like stimuli (“∵”) exert a powerful attention-grabbing effect since early infancy (Buiatti et al., 2019), and possibly even before birth (Reid et al., 2017). Moreover, the oft-cited impairments in decoding facial expression of fear in a patient suffering from amygdala damage (Adolphs et al., 1994) have later been shown to be driven by a failure in the bottom-up attention-grabbing mechanism triggered by faces in healthy subjects (Adolphs et al., 2005).

A few caveats are in order when interpreting the results of our study. First, we want to stress that in our experiment, explanations may have failed to mitigate facial trustworthiness bias because the time constraints we imposed (10 s) were too tight to allow the cognitive control necessary to override biases resulting from facial impressions. In fact, while analysis of response time shows that subjects were significantly slower in assessing explanations as compared to fact, “slower thinking” may not be slow enough for triggering the kind of cognitive control that Kahneman ascribes to the “slow thinking”. Hence, while other studies suggest that such biases are hard to override (see Jaeger et al., 2020), it is an open possibility that replicating our studies with coarser or absent time constraints could yield positive evidence for H2. Similarly, while in our study we find no support for H3, this may be due to the specificity of our sample, which is only composed of Italian subjects. Indeed, several studies have shown how the interpretation of facemasks in terms of trustworthiness can be affected by several factors, e.g. political orientation (Ingram et al. unpublished) or personal distress (Malik et al., 2021; cf. the sub-samples analyzed by Biermann et al., 2021). Hence, it is possible, and rather likely, that masks elicit either trust-enhancing or trust-lowering biases in different samples.

Summing up, while a vast literature attests that facial trustworthiness impression influences observers’ behavior across a variety of contexts, to the best of our knowledge, the present study is the first to show that trustworthiness impression of a given face also biases the credibility of propositional statements associated with it. This result may have relevant implications on several facets of social life – from education to advertising, from science communication to politics. Particular caution seems in order especially when the impression of trustworthiness of some faces is leveraged to deceive.

Moreover, this study further enriches our understanding of the complex social implications of facemasks (Pavlova & Sokolov, 2021): an object that has become commonplace for health-related reasons, yet subtly affecting our social lives also on other, less obvious dimensions. In particular, our study suggests that facemasks may have hitherto unappreciated social implications for their users, not necessarily by impairing the ability to visually detect emotional expressions (Carbon, 2020), but most prominently by changing which information is used as default heuristic in subsequent cognitive processing. This suppression effect need not be considered necessarily negative. Indeed, it may even have positive applications: in contexts in which using visual information on facial features as the ground for action is considered ill-advised or discriminatory, such as trials (Wilson & Rule, 2015) or medical triage (Bagnis et al., 2020), suppressing it may be seen as a desirable outcome, and our results suggest that facemasks may help in that regard. Indeed, facemasks could succeed where neither training (Jaeger et al., 2020) nor cognitive control did (but see the discussion of H2 above). In fact, contrary to what Twele et al., (2022: 16) suggest, they may actually “serve an unintended consequence of creating a more level playing field”, although this equity only holds for certain kinds of games, namely those in which the face needs not be the focus of attention.

## Supplementary Information

Below is the link to the electronic supplementary material.

ESM 1 (DOCX 152 KB)
