# Supplementary material for: Facial impression of trustworthiness biases statement credibility unless suppressed by facemask
Source: Curr Psychol. 2022 Jun 9:1–11. Online ahead of print. doi: 10.1007/s12144-022-03277-7 (PMC9178339; doi:10.1007/s12144-022-03277-7)
Supplement: Supplementary file 1 — (DOCX 152 KB) [file 12144_2022_3277_MOESM1_ESM.docx]

**Supplementary Information**

**Supplementary figures**


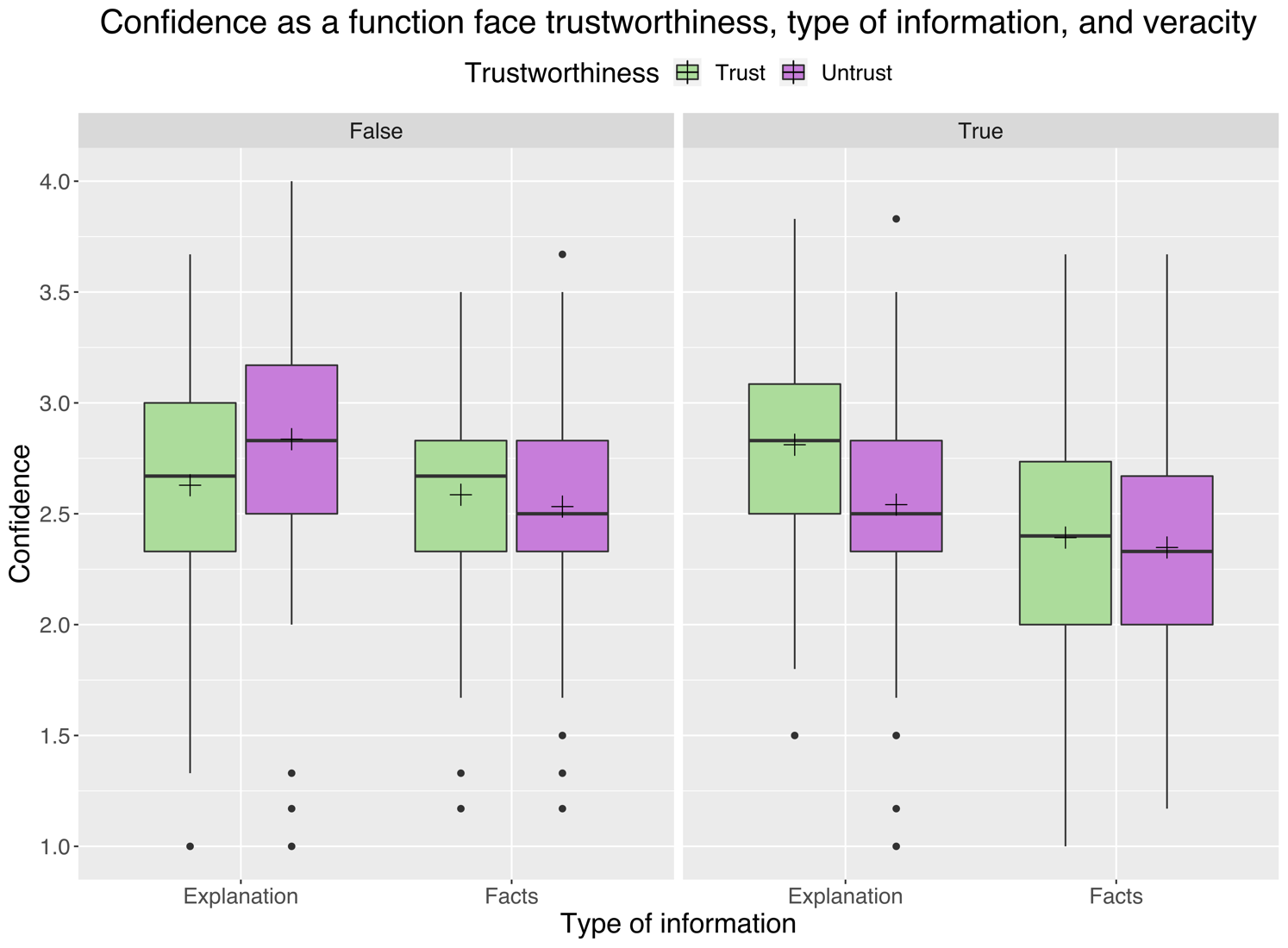


Figure A. Confidence as a function of face trustworthiness impression, type of information, and veracity. The boxplots represent interquartile ranges (IQRs). Black horizontal lines within the boxplots indicate median values; black crosses represent mean values. Black points mark outliers. Trustworthy faces fostered more confidence in all statements. Likewise, greater confidence scores were reported in facts and true statements.

**Stimuli list**

***Faces***

The 48 facial pictures employed in our study were taken from the Chicago Face Database (Ma et al. 2015), balanced for sex and normative trustworthiness rating. In order to eschew confounds due to either racial or age biases, only adult white faces were selected (white being the most widespread ethnicity in Italy).

Trustworthiness scores are based on CFD normative data, varying from 1 to 6.

| Males, High Trustworthiness | | Females, High Trustworthiness | |
| --- | --- | --- | --- |
| CFD id | Avg. Trustworthiness | CFD id | Avg. Trustworthiness |
| WM 014 | 3.80 | WF 011 | 3.70 |
| WM 023 | 3.81 | WF 012 | 3.80 |
| WM 024 | 3.78 | WF 014 | 3.76 |
| WM 029 | 3.70 | WF 030 | 3.79 |
| WM 033 | 3.58 | WF 039 | 3.67 |
| WM 200 | 3.65 | WF 208 | 3.62 |
| WM 202 | 3.76 | WF 211 | 3.89 |
| WM 208 | 3.58 | WF 232 | 3.82 |
| WM 214 | 3.72 | WF 234 | 3.59 |
| WM 225 | 3.70 | WF 235 | 3.72 |
| WM 252 | 3.88 | WF 236 | 3.60 |
| WM 257 | 3.92 | WF 238 | 3.92 |
| *AVG (SD)* | *3.74 (+/- 0.10)* | *AVG (SD)* | *3.74 (+/- 0.10)* |
| Males, Low Trustworthiness | | Females, Low Trustworthiness | |
| CFD id | Avg. Trustworthiness | CFD id | Avg. Trustworthiness |
| WM 019 | 2.69 | WF 201 | 3.09 |
| WM 020 | 3.06 | WF 204 | 3.07 |
| WM 021 | 3.00 | WF 206 | 2.93 |
| WM 038 | 2.96 | WF 210 | 2.65 |
| WM 228 | 3.00 | WF 213 | 3.00 |
| WM 229 | 2.95 | WF 215 | 2.96 |
| WM 232 | 2.77 | WF 222 | 2.89 |
| WM 233 | 3.00 | WF 224 | 3.08 |
| WM 234 | 2.86 | WF 230 | 3.00 |
| WM 237 | 3.00 | WF 239 | 2.83 |
| WM 244 | 2.88 | WF 249 | 3.00 |
| WM 255 | 2.89 | WF 250 | 3.00 |
| *AVG (SD)* | *2.92 (+/-0.10)* | *AVG (SD)* | *2.96 (+/- 0.12)* |

***Sentences***

Below the 48 sentences employed in the Credibility task, sorted for information type (facts / explanation) and veracity (true / false).

Mean length (original): 113 characters; SE = 17.36.

| **True Facts Statements** | |
| --- | --- |
| **Italian (Original)** | **English (Translation)** |
| I pinguini non sono in grado di percepire il gusto dei pesci che mangiano. | Penguins are unable to perceive the taste of the fish they eat. |
| La luna è distante circa 380.000 km dalla Terra. | The moon is about 380,000 km away from Earth. |
| L'olio di oliva raggiunge l'ebollizione ad una temperatura di circa 300°C. | Olive oil reaches the boiling point at a temperature of about 300 ° C. |
| Le dimensioni del nostro fegato cambiano notevolmente fra il giorno e la notte. | The size of our liver changes dramatically between day and night. |
| Il polmone sinistro è del 10% più grande di quello destro, in media. | The left lung is 10% larger than the right, on average. |
| La lunghezza complessiva dei vasi sanguigni presenti in un corpo umano è pari a 3 o 4 volte la circonferenza della Terra. | The total length of the blood vessels present in a human body is equal to 3 or 4 times the circumference of the Earth. |
| I Moai, le caratteristiche statue primitive dell'Isola di Pasqua, possiedono anche un corpo, sepolto nella terra. | The Moai, the characteristic primitive statues of Easter Island, also possess a body, buried in the earth. |
| Nel XVII secolo, molti erano convinti che la California fosse un'isola, nonostante quel territorio fosse colonizzato da tempo. | In the 17th century, many were convinced that California was an island, despite the fact that that territory had been colonized for some time. |
| Durante l'epoca d'oro della pirateria nei Caraibi (1640-1680), l'organizzazione sociale delle ciurme pirata era molto democratica. | During the golden age of piracy in the Caribbean (1640-1680), the social organization of pirate crews was very democratic. |
| Giovanni Segantini fu uno dei principali esponenti della pittura divisionista. | Giovanni Segantini was one of the main exponents of pointillist painting. |
| "Il mito di Sisifo" è un saggio filosofico dello scrittore francese Albert Camus. | "The Myth of Sisyphus" is a philosophical essay by the French writer Albert Camus. |
| Quando Papa Giulio II chiese a Michelangelo di dipingere la volta della Cappella Sistina, la sua prima risposta fu: "Non mi va, sembra un granaio". | When Pope Julius II asked Michelangelo to paint the vault of the Sistine Chapel, his first response was: "I don't want to, it looks like a granary". |

| **False Facts Statements** | |
| --- | --- |
| **Italian (Original)** | **English (Translation)** |
| I lemming, piccoli roditori artici, commettono periodicamente suicidi di massa, gettandosi in mare. | Lemmings, small Arctic rodents, periodically commit mass suicides by jumping into the sea. |
| Il lato della Luna invisibile dalla Terra rimane sempre al buio. | The side of the Moon that is invisible from Earth always remains in the dark. |
| La velocità del suono nell'aria è indipendente dalla temperatura. | The speed of sound in air is independent of temperature. |
| Gli esseri umani normalmente usano solo il 10% del loro cervello. | Humans normally use only 10% of their brains. |
| Tagliare i capelli assicura che poi crescano più resistenti. | Cutting your hair ensures that it then grows more resistant. |
| La percentuale di parti gemellari è inferiore allo 0,5%. | The percentage of twin births is less than 0.5%. |
| L’Italia è diventata uno dei paesi meno industrializzati d’Europa. | Italy has become one of the least industrialized countries in Europe. |
| Tokyo è la città più popolosa del mondo. | Tokyo is the most populous city in the world. |
| La popolazione mondiale al di sotto dei 30 anni di età è pari a circa il 35% del totale. | The world population under the age of 30 is approximately 35% of the total. |
| La Divina Commedia contiene in totale 99 canti. | The Divine Comedy contains a total of 99 *canti*. |
| "La forza del destino" è una famosa opera del compositore italiano Giacomo Puccini. | "The Force of Destiny" is a famous opera by the Italian composer Giacomo Puccini. |
| Il poeta francese Paul Verlaine sparò al suo amico e poeta Arthur Rimbaud, ferendolo mortalmente. | French poet Paul Verlaine shot his friend and poet Arthur Rimbaud, mortally wounding him. |

| **True Explanations** | |
| --- | --- |
| **Italian (Original)** | **English (Translation)** |
| Alcuni animali ingoiano pietre intere che conservano nello stomaco, per favorire la triturazione del cibo. | Some animals swallow whole stones that they store in their stomachs to help break down food. |
| Gli aerei volano perché l’aria sotto le ali si muove più lentamente rispetto all’aria sopra le ali, quindi la pressione dal basso verso l’alto è maggiore. | Airplanes fly because the air under the wings moves slower than the air above the wings, so the pressure from bottom to top is greater. |
| Al tramonto il sole è rosso perché i suoi raggi, attraversando gli strati più bassi dell'atmosfera, perdono alcune frequenze. | At sunset the sun is red because its rays, passing through the lower layers of the atmosphere, lose some frequencies. |
| Quando starnutiamo chiudiamo automaticamente gli occhi per evitare che l'elevata pressione creata dallo starnuto li danneggi. | When we sneeze, we automatically close our eyes to prevent the high pressure created by the sneezing from damaging them. |
| La mattina siamo in media più alti di 1 cm rispetto alla sera, perché la cartilagine fra le nostre ossa tende a comprimersi quando stiamo in piedi a lungo. | In the morning we are on average 1 cm taller than in the evening, because the cartilage between our bones tends to compress when we stand for a long time. |
| Non riusciamo a farci il solletico da soli perché il nostro cervello anticipa la sensazione prodotta dai nostri movimenti, annullando l'effetto sorpresa che rende il solletico divertente. | We can't tickle ourselves because our brain anticipates the sensation produced by our movements, canceling the surprise effect that makes tickling fun. |
| La caduta dell'Impero Romano d'Occidente nel 476 d.C. fu innescata dalla ribellione di mercenari stranieri, grazie ai quali il giovane Romolo Augusto aveva appena conquistato il potere. | The fall of the Western Roman Empire in 476 AD it was triggered by the rebellion of foreign mercenaries, thanks to which the young Romulus Augustus had just conquered power. |
| In epoca Vittoriana si usava fotografare i parenti di un defunto insieme al cadavere, vestito e messo in posa come se fosse vivo, con lo scopo di creare una bella foto ricordo del caro estinto. | In the Victorian era it was customary to photograph the relatives of a deceased together with the corpse, dressed and posed as if it were alive, with the aim of creating a beautiful souvenir photo of the deceased loved one. |
| Nell'antica Roma l'urina raccolta nei bagni pubblici veniva rivenduta e persino tassata, poiché il suo elevato contenuto di ammoniaca la rendeva molto ricercata come detergente. | In ancient Rome, urine collected in public toilets was resold and even taxed, as its high ammonia content made it highly sought after as a cleanser. |
| Si scrive "qualcun altro" senza apostrofo, in quanto si tratta di un troncamento e non di un'elisione. | (In Italian) You write "qualcun altro" without apostrophe, as it is a truncation and not an elision. |
| La prospettiva aerea si chiama così perché riproduce le variazioni di luminosità in rapporto allo strato d'aria tra il pittore e gli oggetti dipinti. | The aerial perspective is so called because it reproduces the variations in brightness in relation to the layer of air between the painter and the painted objects. |
| Il cinese si dice lingua tonale perché pronunciare la stessa sillaba con un tono diverso ne fa variare il significato. | Chinese is called a tonal language because pronouncing the same syllable in a different tone changes its meaning. |

| **False Explanations** | |
| --- | --- |
| **Italian (Original)** | **English (Translation)** |
| Gli oceani liberano grandi quantità di ossigeno nell'atmosfera a causa del movimento delle correnti marine. | The oceans release large amounts of oxygen into the atmosphere due to the movement of sea currents. |
| In estate fa più caldo perché la Terra in quel periodo si trova più vicina al Sole. | In the summer it is warmer because the Earth is closer to the Sun at that time. |
| L'elio contenuto nei palloncini cambia la nostra voce perché indebolisce le corde vocali, che così vibrano in modo diverso. | The helium contained in the balloons changes our voice because it weakens the vocal cords, which thus vibrate differently. |
| Le donne in gravidanza devono evitare il contatto con i gatti per ridurre il rischio che il feto sviluppi allergie. | Pregnant women should avoid contact with cats to reduce the risk of the fetus developing allergies. |
| I capelli diventano bianchi con l'età perché la prolungata esposizione agli agenti atmosferici ne deteriora la pigmentazione. | Hair turns white with age because prolonged exposure to atmospheric agents deteriorates its pigmentation. |
| L'aria di mare ha un effetto positivo sulla salute perché i micro-granuli di sabbia ripuliscono le vie respiratorie e aiutano il filtraggio dei polmoni. | Sea air has a positive effect on health because the micro-grains of sand clean the airways and help filter the lungs. |
| Il cuore veniva lasciato dagli antichi Egizi nel corpo delle mummie perché estrarlo avrebbe rovinato eccessivamente il cadavere. | The heart was left by the ancient Egyptians in the body of the mummies because extracting it would have damaged the corpse excessively. |
| Alcuni monaci buddisti sono riusciti ad auto-mummificarsi, cioè a preservare il proprio corpo dopo la morte senza interventi esterni, grazie a una prolungata esposizione ai fumi di sostanze vegetali, in modo simile all'affumicatura. | Some Buddhist monks have managed to self-mummify themselves, that is, to preserve their body after death without external intervention, thanks to prolonged exposure to the fumes of vegetable substances, similar to smoking. |
| L'epidemia che travolse Atene nel 430 a.C. fu causata dall'avvelenamente dei pozzi del Pireo da parte dei loro storici nemici, gli Spartani. | The epidemic that engulfed Athens in 430 BC it was caused by the poisoning of the wells of Piraeus by their historical enemies, the Spartans. |
| In musica, gli accordi minori si definiscono così perché hanno il minor numero di note. | In music, minor chords are defined this way because they have the fewest notes. |
| La parola "melodia" deriva dalla richiesta dello spartito ("me lo dia!") che i musicisti facevano ai compositori per poter suonare nei concerti a corte. | The word "melody" derives from the request for the score ("me lo dia!", trans. “give it to me!”) that musicians made to composers to be able to play in court concerts. |
| La Monna Lisa di Leonardo si trova al Louvre perché fu sottratta all'Italia durante le spoliazioni napoleoniche. | Leonardo's Mona Lisa is in the Louvre because it was stolen from Italy during the Napoleonic looting. |
